# Predictors of quality of life, functional status, depression and fatigue in early arthritis: comparison between clinically suspect arthralgia, unclassified arthritis and rheumatoid arthritis

**DOI:** 10.1186/s12891-024-07446-6

**Published:** 2024-04-20

**Authors:** Barbara Torlinska, Karim Raza, Andrew Filer, Gurpreet Jutley, Ilfita Sahbudin, Ruchir Singh, Paola de Pablo, Elizabeth Rankin, Benjamin Rhodes, Nicole Amft, Elizabeth Justice, Catherine McGrath, Sangeetha Baskar, Jeanette Trickey, Melanie Calvert, Marie Falahee

**Affiliations:** 1 Centre for Patient-Reported Outcomes Research, Institute of Applied Health Research, University of Birmingham, Birmingham, UK; 2 Birmingham Health Partners Centre for Regulatory Science and Innovation, University of Birmingham, Birmingham, UK; 3 Department of Rheumatology, Sandwell and West Birmingham NHS Trust, Birmingham, UK; 4 Rheumatology Research Group, Institute of Inflammation and Ageing, College of Medical and Dental Sciences, University of Birmingham, Birmingham, B15 2WB UK; 5 NIHR Birmingham Biomedical Research Centre, University of Birmingham, Birmingham, UK; 6 MRC Versus Arthritis Centre for Musculoskeletal Ageing Research and the Research into Inflammatory Arthritis Centre Versus Arthritis, University of Birmingham, Birmingham, UK; 7 Department of Rheumatology, University Hospitals Birmingham NHS Foundation Trust, Birmingham, UK; 8 Department of Rheumatology, Frimley Park Hospital NHS Foundation Trust, Camberley, UK; 9 Department of Rheumatology, The County Hospital, Wye Valley NHS Trust, Hereford, UK; 10 National Institute for Health Research (NIHR) Applied Research Centre West Midlands, Birmingham, UK; 11 Health Data Research UK, London, UK

**Keywords:** Patient-reported outcomes measures, Rheumatoid arthritis, Unclassified arthritis, Clinically suspect arthralgia, Undifferentiated arthritis, Pre-RA stages, Functional status, Health related quality of life, Fatigue, Depression

## Abstract

**Background:**

Rheumatoid arthritis (RA) is often preceded by symptomatic phases during which classification criteria are not fulfilled. The health burden of these “at-risk” stages is not well described. This study assessed health-related quality of life (HRQoL), function, fatigue and depression in newly presenting patients with clinically suspect arthralgia (CSA), unclassified arthritis (UA) or RA.

**Methods:**

Cross-sectional analysis of baseline Patient-Reported Outcome Measures (PROMs) was conducted in patients from the Birmingham Early Arthritis Cohort. HRQoL, function, depression and fatigue at presentation were assessed using EQ-5D, HAQ-DI, PHQ-9 and FACIT-F. PROMs were compared across CSA, UA and RA and with population averages from the HSE with descriptive statistics. Multivariate linear regression assessed associations between PROMs and clinical and sociodemographic variables.

**Results:**

Of 838 patients included in the analysis, 484 had RA, 200 had CSA and 154 had UA. Patients with RA reported worse outcomes for all PROMs than those with CSA or UA. However, “mean EQ-5D utilities were 0.65 (95%CI: 0.61 to 0.69) in CSA, 0.61 (0.56 to 0.66) in UA and 0.47 (0.44 to 0.50) in RA, which was lower than in general and older (≥ 65 years) background populations.” In patients with CSA or UA, HRQoL was comparable to chronic conditions such as heart failure, severe COPD or mild angina. Higher BMI and older age (≥ 60 years) predicted worse depression (PHQ-9: -2.47 (-3.85 to -1.09), *P* < 0.001) and fatigue (FACIT-F: 5.05 (2.37 to 7.73), *P* < 0.001). Women were more likely to report worse function (HAQ-DI: 0.13 (0.03 to 0.21), *P* = 0.01) and fatigue (FACIT-F: -3.64 (-5.59 to -1.70), *P* < 0.001), and residents of more deprived areas experienced decreased function (HAQ-DI: 0.23 (0.10 to 0.36), *P* = 0.001), greater depression (PHQ-9: 1.89 (0.59 to 3.18), *P* = 0.004) and fatigue (FACIT-F: -2.60 (-5.11 to 0.09), *P* = 0.04). After adjustments for confounding factors, diagnostic category was not associated with PROMs, but disease activity and polypharmacy were associated with poorer performance across all PROMs.

**Conclusions:**

Patient-reported outcomes were associated with disease activity and sociodemographic characteristics. Patients presenting with RA reported a higher health burden than those with CSA or UA, however HRQoL in the pre-RA groups was significantly lower than population averages.

**Supplementary Information:**

The online version contains supplementary material available at 10.1186/s12891-024-07446-6.

## Key messages

The development of RA is often preceded by symptomatic phases in which patients do not yet fulfil classification criteria for RA. This study confirms a significant negative impact of symptoms on patient functioning and health-related quality of life in those newly presenting with clinically suspect arthralgia, unclassified arthritis, and RA. Patient burden was associated with measures of disease activity and patient characteristics, and was comparable to serious conditions such as angina, chronic obstructive pulmonary disease and heart failure. Further studies are needed to establish if patients in symptomatic at risk of RA phases may benefit from pharmacological and non-pharmacological interventions to reduce symptom impact and improve quality of life.

**Supplementary Information:**

The online version contains supplementary material available at 10.1186/s12891-024-07446-6.

## Introduction

Rheumatoid arthritis (RA) is a chronic inflammatory joint disease characterised by synovitis, joint destruction, disability, comorbidity and reduced life expectancy [1]. It has a prevalence of 0.5-1% in the UK population and is 2–4 times more common in women than men [2].

RA can be preceded by phases in which patients have inflammatory joint symptoms (with or without clinically apparent synovial swelling) but during which they do not yet fulfil classification criteria for RA. These phases include clinically suspect arthralgia (CSA, symptoms associated with inflammatory arthritis but without clinically apparent synovitis) [3], and unclassified arthritis (UA, clinically apparent synovitis not yet fulfilling criteria for RA or other classifiable forms of arthritis).

Early treatment of RA improves clinical outcomes, including disease activity measures and joint damage [4] and is a key element of treatment guidelines [5]. The stages of disease preceding the development of classifiable RA (also referred to as ‘at-risk’ phases) [6] similarly present opportunities for therapeutic intervention [7, 8]. Most studies assessing the impact of interventions in these ‘at-risk’ phases have assessed impacts on progression to RA. However, interventions in at-risk phases may also benefit patients by modifying risk factors and/or improving symptoms [9].

In order to understand the scope for intervention in symptomatic at-risk phases, it is important to gain a comprehensive understanding of the impact of early symptoms on functioning and quality of life. Subjective perceptions of health status can be captured via patient-reported outcome measures (PROMs), which can quantify symptoms, functional ability and general health status. The impact of RA on PROMs is well understood, and PROMs are included in the core set of measures for RA clinical trials [10, 11]. However, the extent to which PROMs are impacted in pre-RA phases is less clear. Therefore, the aims of this study were (i) to compare PROM scores across patients newly presenting with CSA, UA or RA; (ii) to compare HRQoL reported by patients in these groups with population averages; and (iii) to identify demographic and clinical factors associated with function, HRQoL, fatigue and depression in these patients.

## Methods

### Patient population

This is a cross-sectional analysis of baseline data from the Birmingham Early Arthritis Cohort (BEACON). BEACON recruits adult patients (≥ 18 years) who are naïve to conventional synthetic disease-modifying anti-rheumatic drug (DMARD) therapy and present with either clinically apparent synovitis of ≥ 1 joint or symptoms suggestive of inflammatory arthritis (e.g., morning stiffness, peripheral joint pain, a history of previous joint swelling) in the absence of clinically apparent joint swelling. Patients with symptoms solely due to degenerative joint disease are excluded. Participants are recruited from Sandwell and West Birmingham NHS Trust (SWB), University Hospitals Birmingham NHS Foundation Trust (UHB), and the Modality Partnership. Ethical approval was obtained from the West Midlands – Black Country Research Ethics Committee (Ref 12/WM/0258) and all participants gave written informed consent.

A diagnosis was assigned to each participant following their initial clinical assessment. The current analysis included participants with a diagnosis of CSA, UA or RA between 2013 and 2020. Diagnosis of RA required fulfilment of 1987 ACR criteria [12] or 2010 ACR/ EULAR criteria [13]; a diagnosis of CSA was applied where patients presented with symptoms which, in the opinion of a consultant rheumatologist, were suggestive of inflammatory arthralgia at risk of the development of RA (e.g., morning stiffness, peripheral joint pain, a history of previous joint swelling) in the absence of clinically apparent joint swelling [3]. Patients with UA had clinically apparent synovitis at initial assessment but did not fulfil classification criteria for another inflammatory condition (e.g. RA, psoriatic arthritis, spondyloarthritis, systemic lupus erythematosus, gout, pseudogout, reactive arthritis). Patients with other forms of arthritis or who provided no PROMs assessment were excluded from this analysis. The objectives of the analysis were determined in collaboration with three patient research partners with a diagnosis of RA who reviewed a description of the cohort, the items included in the PROMs, a summary of the data available and a list of potential analytic approaches.

### Demographics and clinical assessment

Data collected at the initial assessment included clinical observations, laboratory findings, demographic data, current medication, and PROMs. Body mass index (BMI) was calculated from weight (kg) and height (m) and was categorised as: underweight and normal weight (< 25 kg/m^2^), overweight (25–30) and obese (≥ 30). Age was categorised into 18–39, 40–49, 50–59, and ≥ 60 years old. For this analysis, ethnicity data were classified as White, Asian, and other, reflecting the major ethnic groups in the area. Smoking status was categorised as current smoker, previous smoker and never smoker. Based on the patient’s residence address, a value of the quintile of the Index of Multiple Deprivation (IMD) [14] was assigned, with a larger number indicating a lower level of deprivation. The middle category (3rd quintile of IMD), reflecting average levels of deprivation, was used as a reference for the linear regression analysis.

Antibody status was recorded as positive if either rheumatoid factor (RF) or anti-cyclic citrullinated peptide (anti-CCP) antibodies were present at the time of first assessment. The Disease Activity Score 28 (DAS-28) was calculated using the C-reactive protein (CRP) formula [15] and categorised as remission (< 2.6), low (≥ 2.6 to < 3.2), moderate (> 3.2 to ≤ 5.1), and high (> 5.1) disease activity [16]. The number of medications used was stratified into < 5 and ≥ 5, based on the most commonly applied definition of polypharmacy [17]. Over-the-counter, topical, herbal, homoeopathic and non-prescribed supplements were excluded.

### Patient reported outcome measures

Study participants completed a range of PROMs at the baseline visit:

#### HAQ-DI

The Stanford Health Assessment Questionnaire Disability Index (HAQ-DI) was specifically developed for the assessment of disability in patients with RA. Patients report the level of difficulty they have in performing certain activities in each of eight functional domains. A Likert scale is used, ranging from 0 (no difficulty) up to 3 (cannot be done at all). Additional scores reflect the use of aids and devices. The final score is the mean of scores of all categories with a range between 0 (best state) and 3 (worst state). HAQ-DI is not computed if the patient did not have scores for at least six categories. HAQ-DI has been shown to be reliable and valid in different languages and contexts [18, 19].

#### EQ-5D

Health-Related Quality of Life (HRQoL) data were collected using EQ-5D, a standardised instrument measuring generic health status. EQ-5D comprises two distinct self-report elements: (i) the health profile: a patient’s self-reported health status on five health dimensions (mobility, self-care, usual activities, pain and anxiety), and (ii) EQ-VAS: a patient’s rating of overall health on a scale from 0 (worst possible) to 100 (best possible health). It is accompanied by a ‘value set’ (also known as ‘utilities’) which reflects the preferences of the general public in a specific population [20]. Utilities can have values from 1 (best health) through 0 (state of health equivalent to death) to the worst state imaginable, which is represented by negative values as it is perceived as a state worse than death.

EQ-5D data were initially collected in the cohort using a 3-level instrument (EQ-5D-3L). This was subsequently replaced by a 5-level instrument (EQ-5D-5L), resulting in approximately a third of the cohort data being assessed with the later version. For the combined analysis, the 5-level data were mapped into the original 3-level utility values [21, 22].

#### PHQ-9

Depression was measured with Patient Health Questionnaire-9 (PHQ-9), a unidimensional depression scale derived from PHQ [23]. A review of the instrument in general, medical, diverse, and rheumatological populations revealed good reliability [24]. It consists of nine items, each of which is scored 0 to 3, indicating the degree of severity (0 - not at all, 1 - several days, 2 - more than half of the days, or 3 - nearly every day), providing a 0 to 27 severity score. To assess the prevalence of depression, PHQ-9 scores were grouped as 0–4 no depression, 5–9 mild depression, and ≥ 10 moderate to severe depression [23].

#### FACIT-F

Evaluation of fatigue in arthritis patients was endorsed by the Outcome Measures in Rheumatology consortium [25]. Fatigue was measured using a unidimensional scale, Functional Assessment of Chronic Illness Therapy – Fatigue (FACIT-F), originally developed for oncology patients with anaemia but subsequently used across a range of chronic illnesses [26]. The measurement covers physical, functional and emotional fatigue as well as social consequences of fatigue. FACIT-F consists of 13 questions rated on a scale of 0–4 (0 – Not at all, 1 – A little bit, 2 – Somewhat, 3 – Quite a bit, 4 – Very much). The final score ranges from 0 to 52, with scores for each of the answers calculated according to the FACIT-F-specific algorithm. When there are missing data, prorating formula ([Sum of item scores] x [N of items] / [N of items answered]) is used as long as more than 50% of the items are answered. Higher scores indicate a lower level of fatigue [27].

### Statistical analysis

Descriptive statistical analyses were performed on complete case data, defined as data available to perform each individual analysis. Continuous data were presented as means and 95% confidence intervals (CI), and categorical data as counts and proportions. Univariate subgroup analyses were performed using t-tests when two groups were compared and ANOVA when there were three or more categories. Post-hoc analysis comparing multiple categories pairwise was conducted applying Bonferroni correction. The correlations between the PROMs of interest were explored by calculating Pearson product-moment correlation coefficient.

A comparison of the HRQoL of diagnostic subgroups with other populations was visualised with a forest plot. The comparison groups encompassed general population data from the Health Survey for England (2017) and subgroups of participants aged 65 years or older and with other common chronic diseases, namely chronic obstructive pulmonary disease (COPD: mild and severe) [28], angina (mild and severe) [29] and heart failure [30].

The univariate analysis was performed independently for each PROM for all factor variables of interest. Variables with *P* > 0.1 were removed from the multivariable model in a backward selection approach, except for diagnosis, which was the variable of interest. The selection of dependent variables was assessed with the likelihood ratio test. Next, the multivariate multiple linear regression model, simultaneously accounting for the correlations between the PROMs, was built using significant variables in at least one of the multivariable models. All analyses were performed using Stata 16.1 (StataCorp LLC, USA). Statistical significance was assumed where *P* < 0.05.

## Results

898 patients in the BEACON cohort had a baseline diagnosis of CSA, UA or RA. Sixty of these patients were excluded as they did not have any PROM data recorded at baseline. The characteristics of excluded patients were largely similar than that of the remaining in the cohort, except for some differences in ethnicity, BMI and diagnosis (Supplementary File, Table 1). The final study group consisted of 838 DMARD-naïve patients diagnosed with either CSA, UA or RA, further reduced in individual analyses due to missing or incomplete outcome data – by 13 (1.5%) in HAQ-DI, 118 (14.1%) in EQ-5D, 26 (3.1%) in FACIT-F and 8 (1.0%) in PHQ-9. Missingness in HAQ-DI, FACIT-F and PHQ-9, ranging between 1 and 3.1%, was assumed unsubstantial. Comparison of patient characteristics with and without valid EQ-5D utilities showed no significant differences between the subgroups (Supplementary File, Table 2).

### Demographic and clinical characteristics with respect to diagnosis

The demographic and clinical characteristics of the study group are presented in Table 1. Patients in at-risk of RA stages constituted nearly half of the study group (23.9% [*n* = 200] with CSA and 18.4% [*n* = 154] with UA); the remaining patients had RA (57.8%, *n* = 484). There were twice as many women as men, and 66.2% were White. 45.3% of patients were living in the areas of the first quintile of IMD (i.e. of highest deprivation). Nearly half never smoked (48.0%), and 22.9% were regularly taking five or more medications.

As expected, diagnostic subgroups differed in the level of disease activity. Over a third of patients with CSA were in DAS-28 remission, while this was the case only in 2.9% of patients with RA. Similarly, only three patients with CSA had a DAS-28 score of > 5.1, whilst nearly a half of those with RA did. There were also significant differences in the proportions of patients with positive antibodies, on polypharmacy and across the categories of duration of symptoms (Table 1).

**Table 1:** Baseline characteristics of study patients, total and by diagnosis subgroups

	Baseline diagnosis			P value	Total (838, 100%)
	CSA (*n* = 200) n (%)	UA (*n* = 154) n (%)	RA (*n* = 484) n (%)		
Female	148(74.0)	87(56.5)	329(68.0)	0.002	564(67.3)
Age					
18–39	74(37.0)	36(23.5)	87(18.0)	< 0.001	197(23.6)
40–49	64(32.0)	42(27.5)	76(15.8)		182(21.8)
50–59	39(19.5)	36(23.5)	143(29.7)		218(26.1)
≥ 60	23(11.5)	39(25.5)	176(36.5)		238(28.5)
Ethnicity					
White	128(66.0)	97(65.1)	317(66.6)	0.4	543(66.2)
Asian	44(22.7)	26(17.4)	92(19.3)		162(19.8)
Other	22(11.3)	26(17.4)	67(14.1)		115(14.0)
BMI					
< 25 kg/m^2^	54(28.3)	39(26.4)	138(30.0)	0.7	231(28.9)
25–30 kg/m^2^	71(37.2)	57(38.5)	154(33.5)		282(35.3)
≥ 30 kg/m^2^	66(34.6)	52(35.1)	168(36.5)		286(35.8)
Smoking status					
Never	104(52.3)	75(49.0)	221(45.9)	0.5	400(48.0)
Current	38(19.1)	33(21.6)	102(21.2)		173(20.7)
Previous	57(28.6)	45(29.4)	159(33.0)		261(31.3)
IMD quintile					
1st (most deprived)	85(42.7)	64(41.6)	230(47.6)	0.5	379(45.3)
2nd	42(21.1)	33(21.4)	107(22.2)		182(21.8)
3rd	37(18.6)	36(23.4)	76(15.7)		149(17.8)
4th	20(10.1)	14(9.1)	34(7.0)		68(8.1)
5th (least deprived)	15(7.5)	7(4.5)	36(7.5)		58(6.9)
DAS-28					
Remission (< 2.6)	72(36.6)	41(27.0)	14(2.9)	< 0.001	127(15.4)
Low (≥ 2.6 to 3.2)	42(21.3)	31(20.4)	24(5.0)		97(11.7)
Moderate (≥ 3.2 to ≤ 5.1)	80(40.6)	71(46.7)	214(44.9)		365(44.2)
High (> 5.1)	3(1.5)	9(5.9)	225(47.2)		237(28.7)
Positive antibodies	87(43.5)	18(11.7)	318(65.7)	< 0.001	423(50.5)
Duration of symptoms					
< 13 weeks	28(14.0)	40(26.5)	124(25.8)	0.002	192(23.1)
13–26 weeks	55(27.5)	40(26.5)	118(24.5)		213(25.6)
27–52 weeks	52(26.0)	28(18.5)	127(26.4)		207(24.9)
≥ 53 weeks	65(32.5)	43(28.5)	112(23.3)		220(26.4)
Polypharmacy					
≥ 5 medications	31(15.5)	28(18.2)	133(27.5)	0.001	192(22.9)

CSA: clinically suspect arthralgia, UA: unclassified arthritis, RA: rheumatoid arthritis, HAQ-DI: severity of disability (increasing), EQ-5D: quality of life (increasing), FACIT-F: fatigue (decreasing), PHQ-9: severity of depression (increasing), BMI: Body Mass Index, IMD: Index of Multiple Deprivation, DAS: Disease Activity Score

### Health state assessment by diagnosis

On average, patients with RA had lower functional status, HRQoL, and worse fatigue and depression than those with CSA or UA. Table 2 presents the mean values of all four PROM measures across the diagnostic groups. There were statistically significant differences between the means, with the RA group consistently having worse outcomes than both CSA and UA groups for all four PROMs (*P* < 0.001 for any pairwise comparison with the RA group), while there were no significant differences between CSA and UA. The prevalence of moderate-to-severe depression was 48% for patients with RA, 32% for those with CSA, and 27% for those with UA.

**Table 2:** Average values of patient-reported outcome measures in patients with clinically suspect arthralgia (CSA), unclassified arthritis (UA), and rheumatoid arthritis (RA). HRQoL: health-related quality of life

	CSA	UA	RA	P value	Post-hoc significant relationship*
Functional status (HAQ-DI)	0.69 (0.60, 0.78)	0.77 (0.66, 0.88)	1.27 (1.20, 1.34)	< 0.001	RA, CSA (< 0.001) RA, UA (< 0.001)
HRQoL (EQ-5D)	0.65 (0.61, 0.69)	0.61 (0.56, 0.66)	0.47 (0.44, 0.50)	< 0.001	RA, CSA (< 0.001) RA, UA (< 0.001)
Fatigue (FACIT-F)	33.5 (31.8, 35.2)	34.9 (33.1, 36.8)	28.7 (27.4, 29.9)	< 0.001	RA, CSA (< 0.001) RA, UA (< 0.001)
Depression (PHQ-9)	7.2 (6.4, 8.0)	6.9 (5.8, 7.9)	10.0 (9.4, 10.6)	< 0.001	RA, CSA (< 0.001) RA, UA (< 0.001)

HAQ-DI: increasing severity of disability, EQ-5D: increasing quality of life, FACIT-F: decreasing fatigue, PHQ-9: increasing severity of depression; * P-value calculated applying Bonferroni correction

**Fig. 1:**
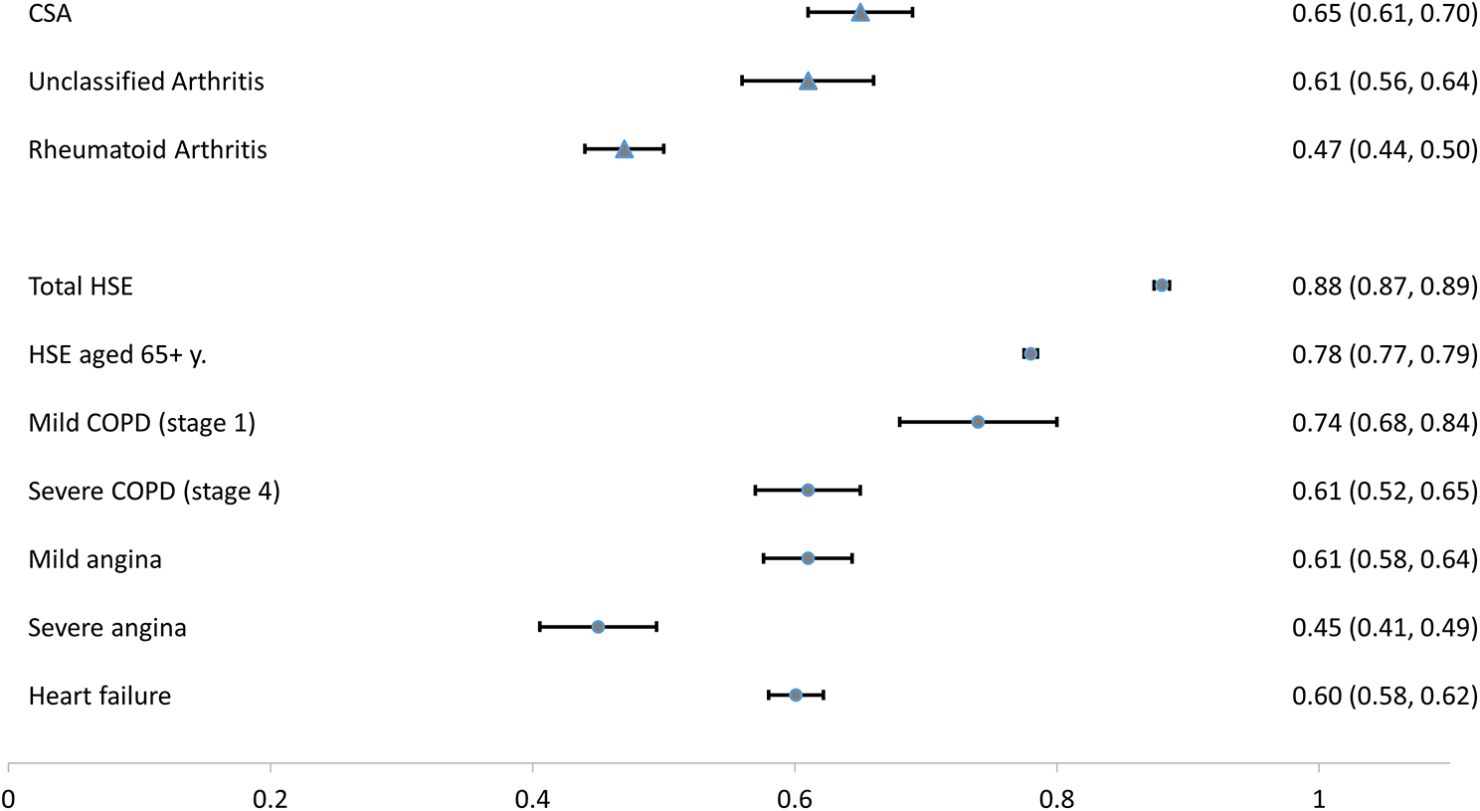
Comparison of Health-Related Quality of Life (HRQoL) expressed as EQ-5D score. Blue triangle: mean EQ-5D in the study diagnostic subgroups; blue circles: mean EQ-5D in the background general and condition-specific populations. Whiskers represent 95% confidence intervals. CSA: clinically suspect arthralgia, HSE: Health Survey for England, COPD: chronic obstructive pulmonary disease

To give context to the HRQoL burden in patients at risk of, or with newly presenting RA, the mean EQ-5D scores in diagnostic groups were compared with the general population and other long-term conditions (Fig. 1). HRQoL across all diagnoses was significantly lower for this cohort than in the Health Survey for England (HSE) population as a total or in a subgroup of participants aged over 65 years. HRQoL in patients with CSA and UA was greater than in those with RA or severe angina and was comparable to patients diagnosed with severe COPD, mild angina or heart failure.

### Associations of the severity of outcomes with clinical and demographic factors

Multivariate analyses were conducted to assess the associations between PROMs (disability, HRQoL, depression and fatigue) with clinical and demographic characteristics; the final model is presented in Table 3. Increased functional disability was associated with female sex, older age, obesity, living in areas with lower quintiles of social deprivation, increased disease activity and polypharmacy. HRQoL was associated with increased disease activity and polypharmacy. The severity of depression (PHQ-9) increased with older age, increasing BMI, living in areas with the lowest quintile of social deprivation, increased disease activity and polypharmacy. Fatigue (FACIT-F) was associated with the female sex, increasing BMI, disease activity and polypharmacy. After adjusting for the demographic and clinical factors, the diagnosis assigned at baseline did not affect any of the studied PROMs. Antibody status, smoking, ethnicity, and duration of symptoms were removed from the model as redundant variables.

**Table 3:** Multivariate analysis of severity of disability (HAQ-DI), quality of life (EQ-5D) and severity of depression (PHQ-9) and fatigue (FACIT-F)

	HAQ-DI			EQ-5D			PHQ-9 score				FACIT-F	
	coef.	(95% CI)	P value	coef.	(95% CI)	P value	coef.	(95% CI)	P value	coef.	(95% CI)	P value
Diagnosis: **CSA**												
UA	0.03	(-0.12, 0.17)	0.72	-0.02	(-0.08, 0.05)	0.61	-0.70	(-2.16, 0.75)	0.34	1.54	(-1.28, 4.36)	0.29
RA	0.03	(-0.11, 0.17)	0.65	0.03	(-0.03, 0.09)	0.36	-0.42	(-1.81, 0.97)	0.55	0.97	(-1.72, 3.67)	0.48
Sex: **Male**												
Female	0.13	(0.03, 0.21)	**0.01**	-0.02	(-0.07, 0.02)	0.29	0.85	(-0.16, 1.85)	0.10	-3.64	(-5.59, -1.70)	**< 0.001**
Age: **18–39 years**												
40–49	-0.02	(-0.17, 0.12)	0.75	0.03	(-0.03, 0.10)	0.27	-0.19	(-1.59, 1.22)	0.79	0.61	(-2.11, 3.33)	0.66
50–59	0.03	(-0.11, 0.17)	0.66	0.02	(-0.04, 0.08)	0.54	-0.43	(-1.79, 0.92)	0.53	2.02	(-0.59, 4.64)	0.13
≥ 60	0.13	(-0.01, 0.27)	0.07	0.04	(-0.02, 0.10)	0.19	-2.47	(-3.85, -1.09)	**< 0.001**	5.05	(2.37, 7.73)	**< 0.001**
Body Mass Index: ** <25 kg/m** ^**2**^												
25–30 kg/m^2^	0.06	(-0.06, 0.18)	0.29	-0.03	(-0.08, 0.02)	0.24	1.26	(0.08, 2.45)	**0.04**	-2.42	(-4.71, -0.13)	**0.04**
≥ 30 kg/m^2^	0.08	(-0.04, 0.20)	0.17	-0.03	(-0.08, 0.03)	0.34	1.41	(0.22, 2.60)	**0.02**	-2.38	(-4.68, -0.08)	**0.04**
Index of Multiple Deprivation: **3rd quintile**												
1st (most deprived)	0.23	(0.10, 0.36)	**0.001**	-0.03	(-0.08, 0.03)	0.35	1.89	(0.59, 3.18)	**0.004**	-2.60	(-5.11, 0.09)	**0.04**
2nd	0.17	(0.02, 0.32)	**0.03**	-0.01	(-0.08, 0.05)	0.71	0.21	(-1.27, 1.70)	0.78	0.12	(-2.75, 3.00)	0.93
4th	0.19	(-0.01, 0.39)	0.05	0.01	(-0.07, 0.10)	0.76	-1.08	(-3.01, 0.85)	0.27	1.19	(-2.54, 4.93)	0.53
5th (least deprived)	0.05	(-0.17, 0.26)	0.68	0.03	(-0.07, 0.12)	0.57	0.01	(-2.09, 2.12)	0.99	0.22	(-3.86, 4.29)	0.92
Disease activity (DAS-28): **Remission**												
Low (≥ 2.6 to 3.2)	0.18	(< 0.01, 0.36)	**0.05**	-0.16	(-0.23, -0.08)	**< 0.001**	0.86	(-0.89, 2.61)	0.34	-0.42	(-3.81, -2.97)	0.81
Moderate (≥ 3.2 to ≤ 5.1)	0.59	(0.44, 0.73)	**< 0.001**	-0.23	(-0.29, -0.17)	**< 0.001**	4.25	(2.79, 5.71)	**< 0.001**	-8.15	(-10.98, -5.31)	**< 0.001**
High (> 5.1)	1.16	(0.98, 1.33)	**< 0.001**	-0.44	(-0.52, -0.37)	**< 0.001**	7.35	(5.59, 9.12)	**< 0.001**	-15.02	(-18.44, -11.61)	**< 0.001**
Polypharmacy: **<5 drugs**												
≥ 5 drugs	0.23	(0.11, 0.34)	**< 0.001**	-0.10	(-0.15, -0.05)	**< 0.001**	1.64	(0.49, 2.79)	**0.005**	-3.43	(-5.66, -1.20)	**0.003**
Constant term	-0.01	(-0.16, 0.22)		0.82	(0.74, 0.91)		3.07	(1.13, 5.02)		42.21	(40.36, 46.78)	

HAQ-DI: increasing severity of disability, EQ-5D: increasing quality of life, FACIT-F: decreasing fatigue, PHQ-9: increasing severity of depression; reference category **in bold**

**Fig. 2:**
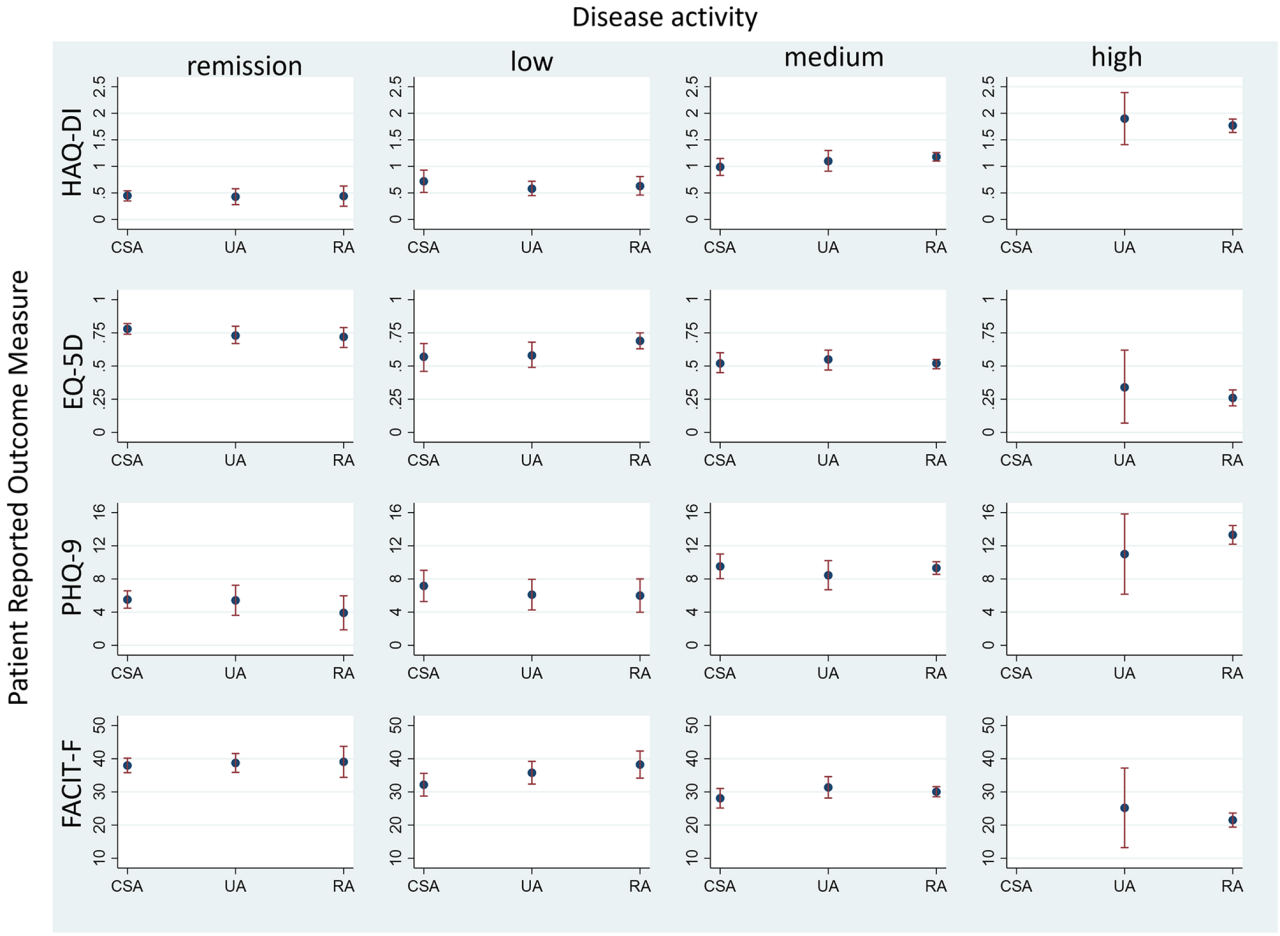
Patient Reported Outcome measures stratified by disease activity in diagnostic subgroups. CSA: clinically suspect arthralgia; UA: unclassified arthritis; RA: rheumatoid arthritis

There were significant differences in the distribution of DAS-28 between the diagnostic groups (Table 1). Figure 2 illustrates the interplay between the diagnosis, disease activity and the studied PROMs. It presents the values of each PROM across all three diagnoses in the DAS-28 strata. In line with the regression model, physical functioning, HRQoL, depression and fatigue were not affected by the diagnosis but varied between DAS-28 states.

## Discussion

This study explores functional impairment (HAQ-DI), health-related quality of life (EQ-5D), depression (PHQ-9), and fatigue (FACIT-F) in patients with CSA and UA, constituting “at-risk” phases of RA, and in patients with newly presenting RA [3]. All disease stages were associated with major negative impacts on patients’ function and well-being, with RA associated with worse scores for all studied PROMs. Comparison of HRQoL with national survey averages revealed significant impairment across all diagnostic subgroups. In CSA and UA, HRQoL was comparable to multiple serious chronic conditions, including severe COPD, mild angina, or heart failure. Patients with RA at initial presentation reported HRQoL comparable to severe angina.

Despite reports of a range of symptoms in the phases preceding RA development [31, 32], there are limited examples of assessments using PROMs in these groups. The extent of functional disability identified in the present study aligns with that found in a longitudinal study of patients with CSA [33]. Interestingly, in those CSA patients who progressed to develop clinical arthritis within a year of follow-up, HAQ scores did not increase between presentation with CSA and arthritis development [34]. A previous study in the Netherlands also reported similar levels of patient burden for CSA as for RA [35].

The relationship between RA status and depression has been shown to be bidirectional: patients with RA are at increased risk of depression, and the incidence of RA is higher in depressed than non-depressed individuals [36]. In patients with early RA, persistent depression and anxiety were also linked with poorer health outcomes over time and reduced treatment response [37]. However, the prevalence of depression has not been well studied in the phases preceding clinically apparent RA. Our findings indicate a significant burden of depression in both CSA and UA patients reflecting an unmet need.

Fatigue is an important feature of inflammatory arthritis and is often described by those affected as one of their most troublesome symptoms [38]. While fatigue is well recognised in RA, it has not been well studied in CSA and UA. The levels of fatigue reported by patients diagnosed with CSA, UA and RA in this study were higher than normative values for the general population [39], indicating increased fatigue in all three diagnostic groups. CSA and UA scores corresponded to the 10–13 percentile of normative data, and RA scores corresponded to the 7–8 percentile, demonstrating that fatigue in pre-RA stages is only slightly less burdensome than in clinically established RA.

Our analysis shows that, on an individual level, patient function and well-being were not associated with baseline diagnosis per se but were strongly affected by disease activity, polypharmacy, obesity and demographic characteristics, including sex, older age, and social status. BMI and, to a lesser degree, social status are modifiable factors affecting patients’ function and well-being. The findings confirm the results of previous studies reporting that unhealthy weight may further exacerbate mental and physical impairments associated with arthralgia or arthritis [40, 41]. Moreover, obesity has also been linked to worse disease activity over follow-up [42–44]. Our data raise the possibility that management of obesity and increased social support in the preclinical stages of the condition may be associated with improved health and well-being.

Our analysis of this large representative sample provides rich insight into important health inequalities. Living in the most deprived areas was associated with a worse functional state and severity of depression but not with overall HRQoL or fatigue. This complements the recent findings of the association of sociodemographic factors with worse disease activity in RA, independently of clinical measures [45]. Research from other countries [46, 47] also links low socioeconomic status with worse PROMs at baseline in patients with early inflammatory arthritis. The inter-relationships between the variables studies in this analysis are consistent with biopsychosocial models of health and disease.

Our study shows that decreased functional status and compromised health outcomes are reported in individuals at risk of RA. Studies of pharmacological intervention in the at-risk stages should therefore assess the impact of the intervention on these important patient-reported outcomes. Furthermore, non-pharmacological approaches, including self-management strategies, are widely recognised in treating patients with RA [48], improving a range of PROMs. The impact of such approaches on patients with CSA and UA also needs to be investigated.

### Strengths and limitations

The main strength of this study is the broad range of validated PROMs, all endorsed by the American College of Rheumatology for use in patients with rheumatic diseases [24, 26, 49, 50], looking at different aspects of patients’ health. The large sample size allowed for complex analysis with high external validity; the sample is socially and demographically diverse and from a cohort with broad inclusion criteria recruited from a diverse catchment area of over 1 million people. Our study includes three major groups often seen in new patient clinics and has important clinical implications, providing insight into often overlooked hidden disabilities, especially in those with CSA where there is no joint swelling and the results of laboratory investigations and imaging may be normal.

Limitations include the recruitment of CSA patients based on clinical opinion. The more recently published set of observations formalising the diagnosis of CSA [3] could not be formally applied as many patients were recruited before these criteria were published and not all relevant variables were collected prior to the point at which the criteria were published. Furthermore, in this cross-sectional analysis, we are unable to relate patient-reported burden at baseline to disease status at follow-up. It should also be noted that causal relationships cannot be inferred from the observed associations.

## Conclusions

Across clinically apparent at-risk stages of RA and in newly presenting RA, patients experience a considerable health burden. Patient-reported function, HRQoL, depression and fatigue were associated with disease activity, polypharmacy, obesity, and demographic factors such as sex, older age and deprivation.

### Electronic supplementary material

Below is the link to the electronic supplementary material.

Supplementary Material 1

## Data Availability

The datasets used and analysed during the current study available from the corresponding author on reasonable request.
