# Supplementary material for: Predictors of quality of life, functional status, depression and fatigue in early arthritis: comparison between clinically suspect arthralgia, unclassified arthritis and rheumatoid arthritis
Source: BMC Musculoskelet Disord. 2024 Apr 20;25:307. doi: 10.1186/s12891-024-07446-6 (PMC11031996; doi:10.1186/s12891-024-07446-6)
Supplement: Supplementary file 1 — Supplementary Material 1 [file 12891_2024_7446_MOESM1_ESM.docx]

**APPENDIX**

**Supplementary Table 1:** Differences between eligible patients included for analysis and those whose PROM questionnaire data were recorded at baseline (excluded from analysis)

|  | **PROMs collected N=838 (%)** | | **No PROMs**  **N=60 (%)** | | **P value** |
| --- | --- | --- | --- | --- | --- |
| Female | 564 | (67.3) | 45 | (75.0) | 0.2 |
| Age (y) |  |  |  |  |  |
| 18-39 | 197 | (23.5) | 18 | (30.0) | 0.3 |
| 40-49 | 182 | (21.7) | 7 | (11.7) |  |
| 50-59 | 218 | (26.0) | 16 | (26.7) |  |
| ≥60 | 238 | (28.4) | 17 | (28.3) |  |
| Ethnicity |  |  |  |  |  |
| White | 543 | (64.7) | 29 | (48.3) | 0.01 |
| Asian | 162 | (19.3) | 20 | (33.3) |  |
| Other | 115 | (13.7) | 6 | (10.0) |  |
| BMI |  |  |  |  |  |
| < 25 kg/m^2^ | 231 | (27.6) | 25 | (41.7) | 0.007 |
| 25-30 kg/m^2^ | 282 | (33.7) | 17 | (28.3) |  |
| ≥ 30 kg/m^2^ | 286 | (34.1) | 10 | (16.7) |  |
| Smoking status |  |  |  |  |  |
| Never | 400 | (47.7) | 29 | (48.3) | 0.5 |
| Current | 173 | (20.6) | 14 | (23.3) |  |
| Previous | 261 | (31.2) | 14 | (23.3) |  |
| IMD quintile |  |  |  |  |  |
| 1^st^ (most deprived) | 379 | (45.3) | 26 | (43.3) | 0.6 |
| 2^nd^ | 182 | (21.8) | 12 | (20.0) |  |
| 3^rd^ | 149 | (17.8) | 13 | (21.7) |  |
| 4^th^ | 68 | (8.1) | 2 | (3.3) |  |
| 5^th^ (least deprived) | 58 | (6.9) | 3 | (5.0) |  |
| Diagnosis |  |  |  |  |  |
| CSA | 200 | (23.9) | 6 | (10.0) | 0.04 |
| UA | 154 | (18.4) | 15 | (25.0) |  |
| RA | 484 | (57.8) | 39 | (65.0) |  |
| DAS 28 (CRP) |  |  |  |  |  |
| Remission (<2.6) | 127 | (15.4) | 9 | (15.0) | 0.5 |
| Low (≥2.6 to 3.2) | 97 | (11.7) | 4 | (6.7) |  |
| Moderate (≥3.2 to ≤5.1) | 365 | (44.2) | 32 | (53.3) |  |
| High (>5.1) | 237 | (28.7) | 15 | (25.0) |  |
| Positive antibodies | 423 | (50.5) | 26 | (43.3) | 0.7 |
| Duration of symptoms |  |  |  |  |  |
| <13 weeks | 192 | (22.9) | 15 | (25.0) | 0.5 |
| 13-26 weeks | 213 | (25.4) | 10 | (16.7) |  |
| 27-52 weeks | 207 | (24.7) | 11 | (18.3) |  |
| ≥53 weeks | 220 | (26.3) | 17 | (28.3) |  |
| Polypharmacy |  |  |  |  |  |
| ≥ 5 medications | 192 | (22.9) | 13 | (21.7) | 0.05 |

CSA: clinically suspect arthralgia, UA: unclassified arthritis, RA: rheumatoid arthritis, HAQ-DI: severity of disability (increasing), EQ-5D: quality of life (increasing), FACIT-F: fatigue (decreasing), PHQ-9: severity of depression (increasing), BMI: Body Mass Index, IMD: Index of Multiple Deprivation, DAS: Disease Activity Score; y: years

**Supplementary Table 2:** Baseline characteristics of subgroups with and without valid EQ-5D score

|  | **Valid EQ-5D N = 720 (%)** | | **No valid EQ-5D**  **N = 118 (%)** | | **P value** |
| --- | --- | --- | --- | --- | --- |
| Female | 485 | (67.4) | 79 | (67.0) | 0.9 |
| Age (y) |  |  |  |  |  |
| 18-39 | 173 | (24.1) | 24 | (20.3) | 0.2 |
| 40-49 | 151 | (21.1) | 31 | (26.3) |  |
| 50-59 | 182 | (25.4) | 36 | (30.5) |  |
| ≥60 | 211 | (29.4) | 27 | (22.9) |  |
| Ethnicity |  |  |  |  |  |
| White | 459 | (65.5) | 83 | (70.3) | 0.6 |
| Asian | 141 | (20.1) | 21 | (17.8) |  |
| Other | 101 | (14.4) | 14 | (11.9) |  |
| BMI |  |  |  |  |  |
| < 25 kg/m^2^ | 200 | (29.1) | 31 | (27.9) | 0.6 |
| 25-30 kg/m^2^ | 238 | (34.6) | 44 | (39.6) |  |
| ≥ 30 kg/m^2^ | 250 | (36.3) | 36 | (32.4) |  |
| Smoking status |  |  |  |  |  |
| Never | 351 | (49.0) | 49 | (41.5) | 0.3 |
| Current | 146 | (20.4) | 27 | (22.9) |  |
| Previous | 219 | (30.6) | 42 | (35.6) |  |
| IMD quintile |  |  |  |  |  |
| 1^st^ (most deprived) | 326 | (45.4) | 53 | (44.9) | 0.5 |
| 2^nd^ | 153 | (21.3) | 29 | (24.6) |  |
| 3^rd^ | 134 | (18.7) | 15 | (12.7) |  |
| 4^th^ | 58 | (8.1) | 10 | (8.5) |  |
| 5^th^ (least deprived) | 47 | (6.6) | 11 | (9.3) |  |
| Diagnosis |  |  |  |  |  |
| CSA | 167 | (23.2) | 33 | (28.0) | 0.2 |
| UA | 128 | (17.8) | 26 | (22.0) |  |
| RA | 425 | (59.0) | 59 | (50.0) |  |
| DAS 28 (CRP) |  |  |  |  |  |
| Remission (<2.6) | 109 | (15.3) | 18 | (15.8) | 0.08 |
| Low (≥2.6 to 3.2) | 88 | (12.4) | 9 | (7.9) |  |
| Moderate (≥3.2 to ≤5.1) | 303 | (42.6) | 62 | (54.4) |  |
| High (>5.1) | 212 | (29.8) | 25 | (21.9) |  |
| Positive antibodies | 372 | (51.7) | 51 | (43.2) | 0.09 |
| Duration of symptoms |  |  |  |  |  |
| <13 weeks | 160 | (22.4) | 32 | (27.4) | 0.5 |
| 13-26 weeks | 181 | (25.3) | 32 | (27.4) |  |
| 27-52 weeks | 181 | (25.3) | 26 | (22.2) |  |
| ≥53 weeks | 193 | (27.0) | 27 | (23.1) |  |
| Polypharmacy |  |  |  |  |  |
| ≥ 5 medications | 169 | (23.5) | 23 | (19.5) | 0.3 |

CSA: clinically suspect arthralgia, UA: unclassified arthritis, RA: rheumatoid arthritis, HAQ-DI: severity of disability (increasing), EQ-5D: quality of life (increasing), FACIT-F: fatigue (decreasing), PHQ-9: severity of depression (increasing), BMI: Body Mass Index, IMD: Index of Multiple Deprivation, DAS: Disease Activity Score; y: years
